# New protocol for optimisation of polymer composition for imprinting of peptides and proteins

**DOI:** 10.1039/c9ra05009d

**Published:** 2019-09-04

**Authors:** Thomas S. Bedwell, Nadeem Anjum, Yifeng Ma, Joanna Czulak, Alessandro Poma, Elena Piletska, Michael J. Whitcombe, Sergey A. Piletsky

**Affiliations:** Department of Chemistry, University of Leicester LE1 7RH UK sp523@le.ac.uk; Division of Biomaterials and Tissue Engineering, UCL Eastman Dental Institute 256 Gray's Inn Road London WC1X 8LD UK

## Abstract

We present here a novel screening tool for optimisation of polymerisation mixtures used in imprinting of peptides and proteins. To facilitate rapid synthesis and screening of a combinatorial library of polymers the solid-phase synthesis method developed by Piletsky and co-workers was scaled down to 50 mg of template-immobilised solid phase, allowing a single well of a 96-well microplate to function as an individual reaction vessel. In this way, 32 different polymer compositions containing *N*-isopropylacrylamide, acrylic acid, *N*-(3-aminopropyl)methacrylamide hydrochloride, and *N-tert*-butylacrylamide, were tested in imprinting of three peptides and three proteins. Utilising filtration microplates has allowed the elution and washing steps to be performed in a similar manner to the large-scale synthesis, whilst incorporation of a fluorescent monomer (*N*-fluoresceinylacrylamide) made it possible to analyse the binding of synthesised polymer nanoparticles to the solid phase with immobilised templates under different washing conditions. The experiment has proven that the variations in monomer compositions had an effect on the yield and affinity of synthesised molecularly imprinted polymers for the peptides, but not for the proteins. Imprinting in this way presents an ideal method for performing small-scale syntheses for testing polymerisation mixtures, as information regarding the molecularly imprinted polymers affinity can be assessed as part of the elution process, without a need for time-consuming analysis such as quartz crystal microbalance or surface plasmon resonance.

## Abstract 2

A novel screening tool for high-throughput optimisation of monomer composition for imprinting of peptides and proteins.

## Graphical abstract

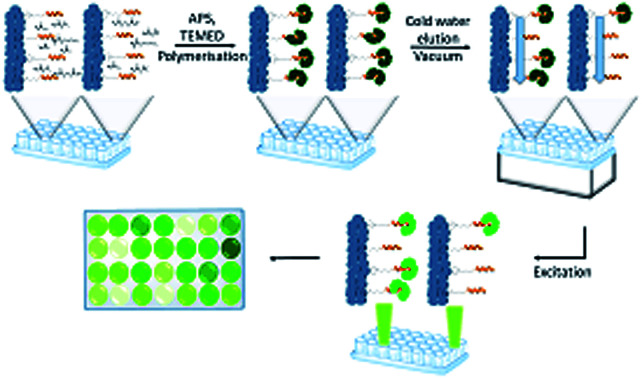

## Introduction

Specific receptor–ligand interactions are an intrinsic part of biological machinery and essential for the generation of physiological responses to hormones, proteins, cellular markers, antigens *etc.* The specific nature of biological recognition, in particular of antibodies and enzymes, has led to their exploitation as the recognition element in assays and sensors. However, despite possessing high specificity and sensitivity for their respective ligands, biomolecules suffer from disadvantages such as fragility and high manufacturing costs. Consequently, much effort has been invested into design and synthesis of artificial materials with biomimetic properties, such as molecularly imprinted polymers (MIPs). Coupled with the advantages of short synthesis time, robustness, regeneration (and consequently cost efficiency), as well as cheap initial production, MIPs provide an attractive alternative to conventional biological receptors. As a result, molecular imprinting has been utilised in a number of applications, including purification and separation, sensing, catalysis, drug delivery, and in a variety of assays and sensors.^1–9^

A series of significant breakthroughs in MIP technology came as a result of novel synthetic methods to generate spherical, molecularly imprinted beads as an alternative to conventional MIP particles produced through bulk polymerisation followed by grinding into small particles.^10,11^ Nanoparticles in particular offer strong advantages to conventional, bulk MIPs, such as low level of nonspecific binding, quick binding kinetics, and adaptable protocols for replacing antibodies and enzymes in assays and sensors. As a next step in advancing this technology Poma *et al.* has developed a method for solid-phase synthesis of MIP nanoparticles (nanoMIPs) with “monoclonal” binding properties, suitable for automation in a computer-controlled reactor.^12,13^ This further cemented benefits of molecular imprinting over alternatives, such as antibodies, aptamers and biosimilars, by offering a convenient way for large-scale and low cost production of MIPs. Development of nanoMIPs is shorter and less expensive than antibody development, experimental animals are not involved in the process, and MIPs do not require cold storage and cold-chain logistics.

The molecular imprinting technology is not however without limitations. Barriers to adoption of this new technology may be uncertainty over security of supply and the perception that changes need to be made in manufacturing practices and plant in order to make the switch from antibodies to MIPs. Particularly challenging remains the imprinting of large macromolecules such as proteins, glycoproteins and nucleic acids. The difficulties are linked with the selection of monomer compositions suitable for imprinting of water-soluble biological macromolecules, and are a consequence of the large size and structural complexity of these targets resulting in steric and conformational issues, as well as the aqueous environment having a dramatic effect on the interactions required for binding.^14,15^ Reports of protein imprinting in the literature have greatly increased in recent years; however, as mentioned, proteins are difficult templates to work with and not all reports provide strong evidence for imprinting. Kryscio *et al.* have shown that the structure of proteins typically employed as templates are adversely affected by exposure to monomers commonly used in imprinting.^16,17^ Verheyen and co-workers have also highlighted the problems of nonspecific interactions with polymers carrying charged monomers, which can overwhelm specific binding to MIPs.^18^ The overwhelming conclusion points to epitope imprinting as an obvious solution free of many of the pitfalls associated with imprinting of macromolecules.^19^ Peptides occupy an interesting middle ground between small molecules and proteins. Akin to small molecules, they possess a more rigid structure, unlikely to take on any secondary conformation. Representing a simpler system than a protein, optimisation of polymer composition for peptides may help to bridge the gap between small molecule and protein imprinting.

The defining property underpinning the success or failure of molecular recognition is the strength of the complementary interactions between the functionalities presented by the analyte and receptor. In the case of a MIP, these functionalities are introduced through the monomers, the selection of which is therefore crucial to maximising the ability of a polymer to bind the desired template. The optimisation of monomer compositions is however a very time consuming process, with lab-based approaches focusing on combinatorial synthesis and screening.^20,21^ With vast quantities of functional monomers either commercially available or readily synthesised, narrowing the selection to those most optimal for a particular target is a daunting task. The advent of *in silico* tools to aid in this process is therefore welcomed, and coupled with a rapid lab-based screen has the potential to produce far superior MIPs to those synthesised using typical compositions without investing a large amount of time. So far however computational design of polymeric adsorbents has proven to be more successful for small molecules than for peptides and proteins.^22^ The reason for this lies in small variations in the structure and a large number of polar domains in a large protein that cannot be discriminated by virtually designed polymers in molecular modelling experiments.^23^ Unfortunately it is very difficult to model hydrophobic interactions, which otherwise would be useful for the design of MIPs for an aqueous environment. In addition, the requirement to perform imprinting in an aqueous environment limits the number of monomers available for polymerisation. Whilst some of these monomers are simply not soluble in water, a more important consequence of using this solvent lies in the diminished effect of electrostatic interactions in water.^24^

**Fig. 1:**
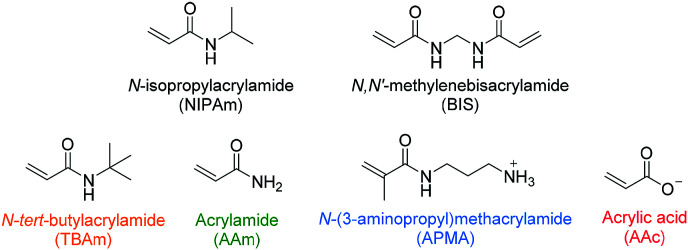
Monomers used by Hoshino *et al.* in combinatorial library preparation.^25^ Hydrophobic, hydrogen bonding and positive/negative charged functional monomers are indicated in orange, green, blue/red.

In recent literature the selection of functional monomers for protein imprinting is rarely commented upon, with little optimisation of polymer compositions appearing to be performed. This may be due to the lack of an established method for optimisation of monomer composition. In one such rare paper Shea and co-workers have used combinatorial screening of a polymer library to develop nanoMIPs capable of recognising melittin.^25^ The authors have systematically varied the composition of functional monomers for the synthesis of polymer nanoparticles, which were then screened for their affinity to the target molecule. The selected monomers consisted of six acrylamide derivatives: the bulk of each polymer consisted of *N*-isopropylacrylamide (NIPAm) as backbone monomer in combination with acrylamide, acrylic acid (AAc), *N*-(3-aminopropyl)methacrylamide hydrochloride (APMA), and *N-tert*-butylacrylamide (TBAm), as hydrogen bonding, negative-charged, positive-charged, and hydrophobic functional monomers, as well as *N*,*N*′-methylenebis(acrylamide) (BIS) (2 mol%) as a cross-linker (Fig. 1). Of the 13 polymers synthesised, only two compositions showed appreciable affinity towards the target when analysed *via* quartz crystal microbalance (QCM), demonstrating the importance of optimising monomer composition to achieve successful imprinting.

The aim of this work is to develop a convenient protocol for screening the ability of functional monomers to form high performance nanoMIPs for peptides and proteins. The small library used in the work of Hoshino *et al.*^25^ provided a starting point for our study. In contrast to this previous work nanoMIPs were produced by a solid-phase approach in our study.^13^ This approach represents an ideal method for performing small-scale syntheses for testing polymer compositions, as information regarding the MIPs affinity can be assessed as part of the elution process.^20^

## Experimental

### Materials and methods

All chemicals used were purchased from commercial sources and used without further purification unless otherwise stated. All chemicals were stored under conditions outlined in the manufacturer's instructions. All peptides were purchased from Bachem with >95% purity, whilst all proteins were sourced from Sigma-Aldrich, UK. Spheriglass A-Glass 2429 microspheres used as solid phase for template immobilisation were obtained from Blagden Chemicals, UK. 300 μL polypropylene 96-well filtration microplates (25 μm polyethylene membranes) and vacuum manifold utilised for small-scale polymer composition screening were purchased from Porvair Sciences, UK.

Fluorescence microplate measurements were performed using a Hidex Sense microplate reader. Optical setups were optimised dependent upon the fluorophore/chromophore and concentrations used.

### Activation of glass microspheres

Glass microspheres (200 g) were boiled in sodium hydroxide (4 M, 160 mL) for 15 minutes prior to washing with 3 volumes (500 mL) of water. The beads were subsequently placed in a solution of sulphuric acid (50%, 160 mL) for 30 minutes before again washing with water (500 mL) and phosphate buffered saline (PBS, 500 mL), ensuring the final pH is between 6–8. Further washing with acetone (500 mL) was performed before drying under vacuum and placing the beads in an oven (150 °C) for 30 minutes.

### Silanisation of glass microspheres

Activated glass microspheres (200 g) were incubated in a solution of toluene (80 mL) with (3-aminopropyl)triethoxysilane (1.6 mL) and 1,2-bis(triethoxy-silyl)ethane (0.270 mL) overnight at 70 °C. Beads were subsequently washed with 3 volumes of methanol and 5 volumes of acetone to remove any residual silane, before drying under vacuum and further oven drying for 30 minutes at 150 °C.

### Immobilisation of peptides

Succinimidyl iodoacetate (SIA) (10 mg) was added to silanised solid phase (120 g) in anhydrous acetonitrile (50 mL) and incubated for 2 h under exclusion of light, before washing with acetonitrile (5 × 50 mL). Thiol buffer (pH 8.2) consisting of PBS (50 mL, 10 mM) and ethylenediaminetetraacetic acid (74 mg) was degassed and purged with nitrogen prior to addition of peptide (5 mg). Incubation with SIA-functionalised solid phase (120 g) was allowed overnight with exclusion of light, followed by washing with water (1.5 L) and drying under vacuum.

### Immobilisation of proteins

Silanised solid-phase (40 g) was incubated in a solution of glutaraldehyde (1.12 mL) in PBS (16 mL, pH 7.4) for two hours before washing with water (8 × 16 mL). Glutaraldehyde-functionalised solid phase (40 g) was incubated in a solution of protein (8.0 mg) in PBS (16 mL, pH 7.4) for 1 hour before washing with water (12 × 16 mL). Protein-immobilised solid phase was stored at −20 °C.

### Solid phase synthesis of MIP NPs

50 mg of solid phase was used per synthesis. The quantities given below are therefore those employed for a typical synthesis.

Polymerisation mixture consisting of NIPAm (39 mg, 344.64 μmol), BIS (2 mg, 12.97 μmol), TBAm (33 mg, 259.47 μmol dissolved in 1 mL ethanol), AAc (100 μL of a 22 μL mL^−1^ solution in water, 31.92 μmol), APMA (5.80 mg, 33 μmol), and if fluorescent MIPs are desired, *N*-fluoresceinylacrylamide (2.5 mg dissolved in 1 mL ethanol), was dissolved in water (100 mL) and purged with nitrogen for 30 minutes. Following this, the polymerisation mixture was added to template-derivatised beads (60 g) and polymerisation initiated using a solution of ammonium persulfate (APS, 30 mg/500 μL water, 131.47 μmol) and *N*,*N*,*N*′,*N*′-tetramethylethylenediamine (TEMED, 30 μL, 70.03 μmol). The polymerisation was allowed to proceed for 60 minutes, before quenching of the reaction by allowing oxygen into the system. The beads were subsequently washed with water (9 × 30 mL) at room temperature to remove unreacted monomer and low affinity polymer before eluting high-affinity nanoparticles with hot water (100 mL) at 60 °C.

### Small-scale synthesis screen with filtration microplates

The standard polymerisation protocol was adapted to be performed in a single well of a 96 well microplate. Each polymerisation mixture (1 mL) was prepared with the functional monomer compositions modified as outlined in Table 1. These were then dispensed (100 μL per well) in triplicate into wells containing functionalised solid phase (50 mg), before initiating the polymerisation with APS and TEMED and leaving for 1 hour at room temperature. The monomer solution was then removed from each well by fitting the microplate into a vacuum manifold, and the solid phase washed with water (10 × 100 μL) to remove unreacted monomer and low-affinity polymer. After fluorescence measurements, further washing was performed in the same fashion using water at 60 °C to emulate the elution process of high-affinity MIPs, before again taking fluorescence measurements.

**Table 1:** Ratio of functional monomers used in nanoMIPs

Entry	Functional monomers ratio^a^ (mol%)		
	TBAm	AAc	APMA
1	40	0	5
2	40	10	5
3	40	15	5
4	40	5	0
5	40	5	10
6	40	5	15
7	0	5	5
8	55	5	5
9	65	5	5
10	40	5	5

a Mol% made up to 100% with NIPAm.

The quantity of MIP still bound to immobilised template was measured using the fluorescence introduced to the polymers through *N*-fluoresceinylacrylamide. A filter set with excitation of 485/10 nm and emission of 520/14 nm was used, with a dichroic mirror at 505 nm.

### Analysis of the size of MIP NPs

Nanoparticle size was determined by DLS using a Zetasizer Nano (Nano-S) from Malvern Instruments Ltd. (Malvern, UK). Prior to DLS measurements samples were subjected to sonication and vortexing before filtering through a 1.2 μm glass fibre syringe filter. All measurements were performed at 25 °C.

### MIP affinity measurements by SPR

SPR experiments were performed using a BIAcore 3000 (GE Healthcare). Bare gold sensor chips were incubated overnight with 11-mercaptoundecanoic acid (22 mg in ethanol (10 mL)) to afford a carboxyl-functionalised surface and were rinsed with ethanol and dried under nitrogen immediately before use. All MIPs were immobilised using amine-coupling chemistry at a flow rate of 5 μL min^−1^. The surfaces of flow cells one and two were activated with 35 μL of a 1 : 1 mixture of *N*-hydroxysuccinimide (0.1 M) and *N*-(3-dimethylaminopropyl)-*N*′-ethylcarbodiimide hydrochloride (0.4 M). MIPs (35 μL, 10–200 μg mL^−1^ in 10 mM sodium acetate, pH 5.0) were then immobilised on flow cell 2, with a control polymer immobilised on flow cell 1 to serve as a reference surface immobilisation responses matched as closely as possible. Both surfaces were subsequently blocked with a 7 min injection of ethanolamine (1 M, pH 8.0). To collect kinetic binding data analyte was injected over both flow cells at a rate of 15 μL min^−1^ at 25 °C, using PBS as running buffer and for all analyte dilutions. A kinetic titration injection strategy was employed, with analyte allowed to associate and dissociate for 14 and 5 min respectively, before a final dissociation of 120 min. All data were fit to a 1 : 1 interaction model using BIAevaluation software, with chi^2^ values used to determine the goodness of fit.

## Results and discussion

To facilitate rapid synthesis and screening of a combinatorial library of polymers the solid-phase synthesis method was scaled down to 50 mg of solid phase with immobilised template, allowing a single well of a 96-well microplate to function as an individual reaction vessel. In this way, 32 different polymer compositions can be simultaneously produced and tested. Filtration microplates allow the elution and washing steps to be performed in a similar manner to the large-scale synthesis, whilst incorporation of a fluorescent monomer makes it possible to analyse binding of nanoMIPs to the immobilised template under different washing conditions. The monomer mixture was adapted from that of Hoshino *et al.* (Table 1).^25^ Each composition contained a small quantity (1%) of *N*-fluoresceinylacrylamide for monitoring of nanoMIPs binding by fluorescence measurements.

**Fig. 2:**
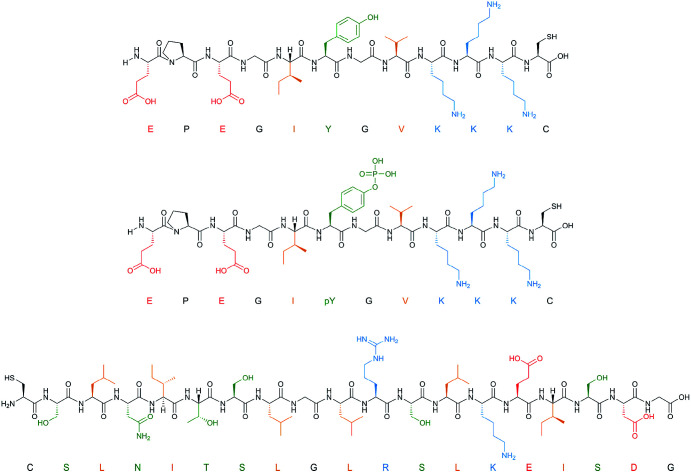
Peptide templates employed for composition screening. Acidic (red), basic (blue), polar (green) and hydrophobic (orange) residues are all highlighted.

Three proteins (amylase, albumin, and trypsin) and three peptide targets were investigated using the proposed screening method (Fig. 2). Two of the selected peptides have identical sequences differing only through their phosphorylation state; the parallel structures were chosen as a way to assess the selectivity introduced through different polymer compositions for chemically similar structures. The third peptide was chosen to be distinctly different to the others to observe how much influence the amino acid sequence would have on the polymer composition.

Solid phase was prepared in bulk before dividing into 50 mg aliquots for direct addition to each well of the filtration microplate. Synthesis of nanoMIPs was performed as described earlier.^26^ Briefly, this involved activation of silica beads through boiling in sodium hydroxide, prior to silanisation to afford free amine groups on the surface for functionalisation. SIA was subsequently used to couple the free amines to the terminal cysteines of the peptides, achieving site-specific immobilisation of each peptide in a fixed orientation. Proteins were immobilised onto glass beads by glutaraldehyde chemistry.

Having loaded peptide-immobilised solid phase into the wells of a filtration microplate, the prepared monomer solutions were added and polymerisation initiated chemically through addition of APS and TEMED. After an hour the unreacted monomer and low affinity polymer were removed by vacuum filtration, and the solid phase washed with 10 volumes of room temperature water (no appreciable drop in fluorescence was observed with additional washes), before measuring the fluorescence. The fluorescence after the cold washes is a result of the “high affinity” MIPs still remaining on the solid phase. No appreciable increase in fluorescence was observed after two hours polymerisation, indicating that 1 hour is sufficient time to form high affinity nanoMIPs.

From measuring the fluorescence of nanoMIPs bound to the solid phase with immobilised target, we can conclude that regardless of polymer composition a significant quantity of nanoparticles remain bound to the solid-phase. This is in contrast to the work carried out by Hoshino *et al.*, in which very few polymer compositions demonstrated appreciable affinity. This can be rationalised by comparing the two synthetic methods; in Hoshino's experiments, MIPs were prepared by precipitation polymerisation with no affinity separation step. A sample of MIPs produced in this way will therefore demonstrate a wide distribution of affinities for the template, with the response observed by a method such as QCM representing the average affinity of all particles in the sample. In solid-phase synthesis, however, MIPs with low affinity are discarded—whilst this results in lower polymer yield, the nanoparticles produced will have a narrower distribution and higher affinity.

**Fig. 3:**
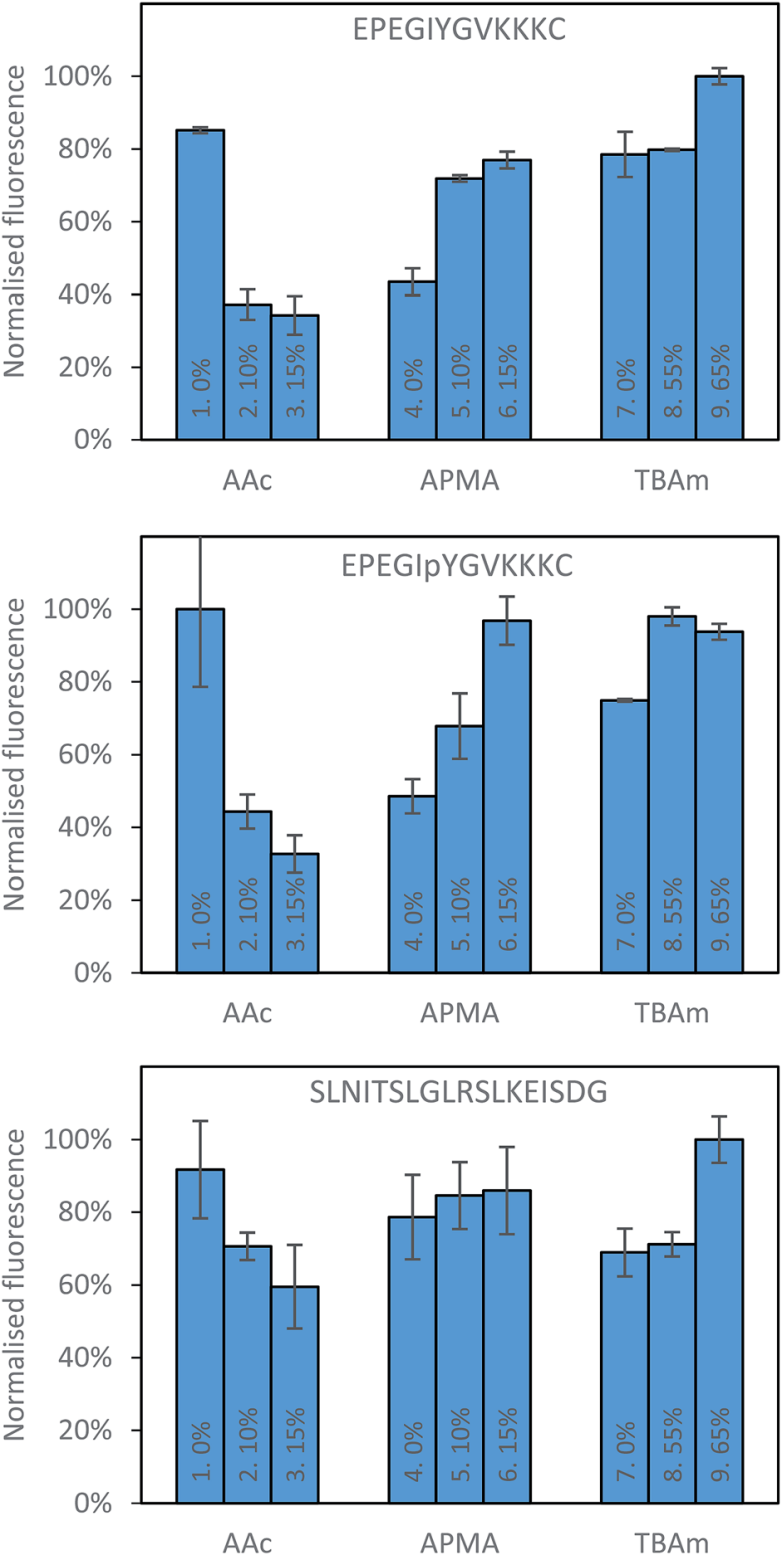
Percentage bound for each polymer composition against all three peptides following washing with 10 volumes of room temperature water.

There are also clear trends that can be identified from the data collected after washing of the solid phase (Fig. 3). It appears that for each peptide, the exclusion of AAc is beneficial. This is not immediately obvious why its inclusion would be detrimental to the polymer binding, since there are residues in all peptides that can form electrostatic interaction with AAc, especially in case of SLN-, which has positively charged lysine and arginine residues. A possible interpretation is that AAc and APMA in the monomer mixture tend to interact with each other, which prevents them from interacting with the template. The very strong negative impact AAc had on phosphorylated peptide can be explained by the electrostatic repulsion between the negative charges of the monomer and the template. This suggests that in general, for peptide imprinting, AAc is not beneficial, however with an increased number of basic residues it may become advantageous. For APMA the opposite trend is observed for all three peptides, where increasing the concentration of these functional monomers results in a greater retention of polymer (although not significantly in the case of SLN-). All three peptides contain negatively charged glutamic acid residues, and so increasing concentration of APMA being favourable is logical. The benefit is most drastic in the phosphopeptide, indicating that the introduction of additional negative charge through the phosphate group promote interactions with the APMA in the polymer. For TBAm the positive trend is observed for all three peptides, especially for non-phosphorylated EPE – where increasing the concentration of these functional monomers results in a greater retention of polymer. Again, all three peptides contain hydrophobic residues, and so TBAm being beneficial is of no surprise. Overall, the combinatorial approach presented here will allow rational exploitation of both hydrophobic and electrostatic interactions.^27^

**Fig. 4:**
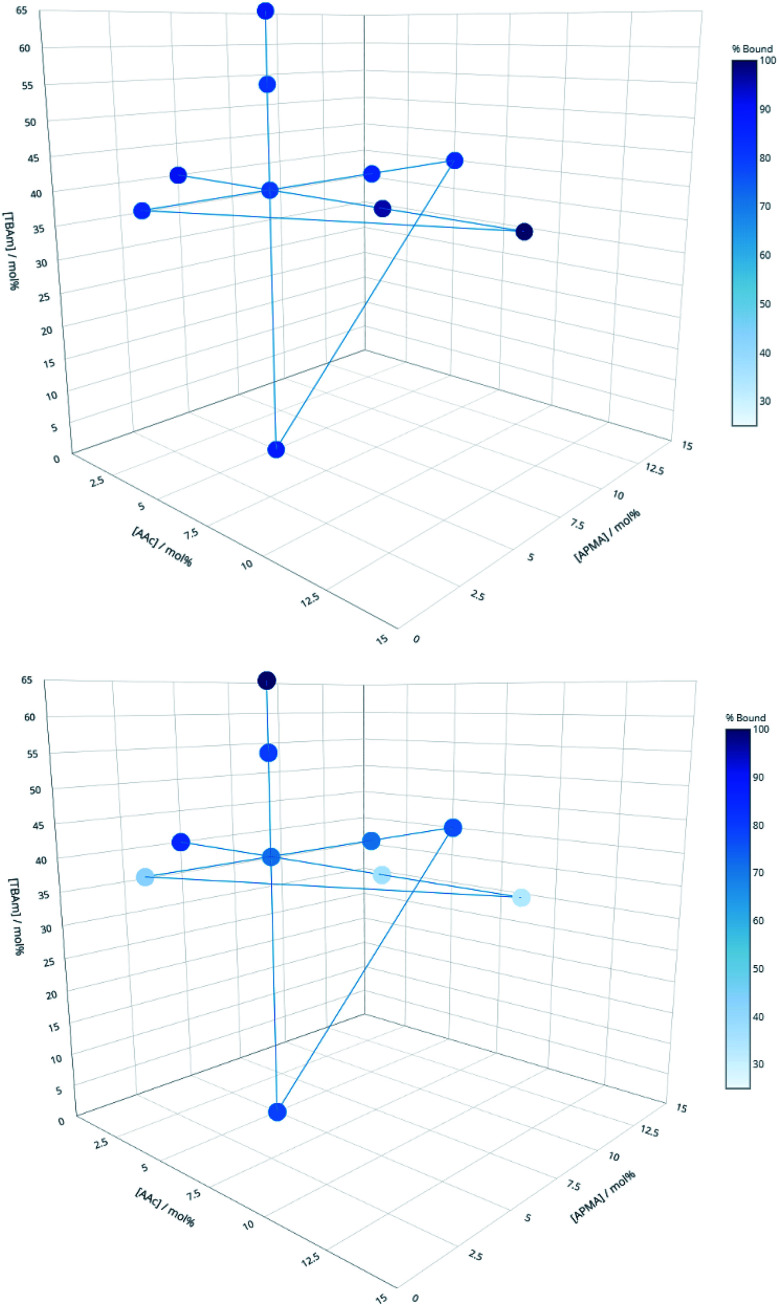
Percentage of polymer retained for each monomer composition for albumin (top) and EPEGIYGVKKKC (bottom).

Three proteins; amylase, albumin, and trypsin were investigated in the same manner as the aforementioned peptides. The immediate observation from this data is how little influence the polymer composition has on the retention of MIPs compared to the experiments conducted against peptides (Fig. 4).

**Fig. 5:**
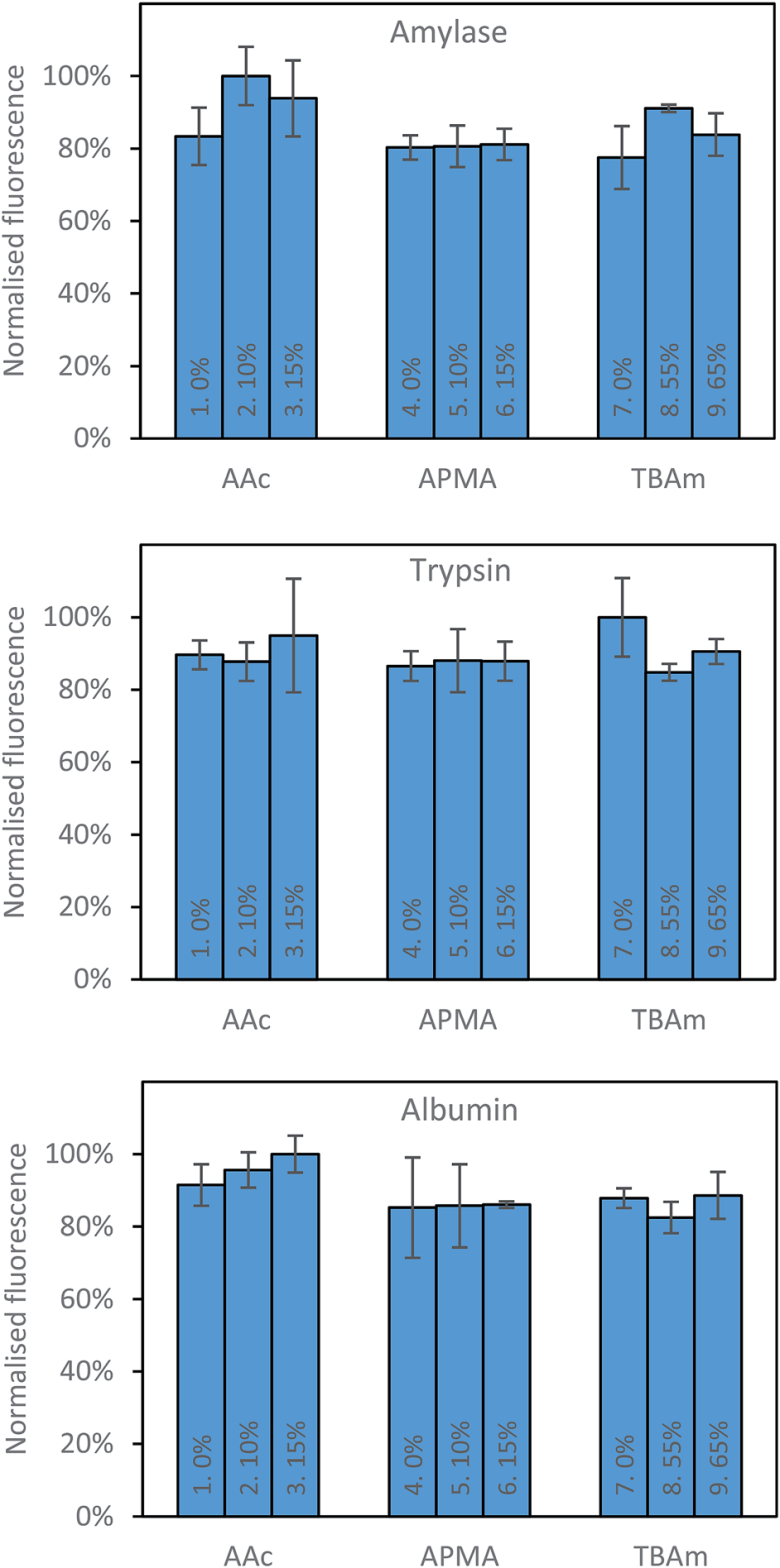
Percentage bound for each polymer composition against all three proteins following washing with 10 volumes of room temperature water.

For all of the compositions tested, the difference in response was no greater than 20% (Fig. 5). This is not particularly surprising; during the imprinting of such a large template with an abundance of interaction sites available for monomers to pre-assemble themselves around, it would be very unlikely that the inclusion/omission of a particular monomer would result in an inability to generate a high affinity interaction with any of these points of interaction. In contrast to the imprinting of peptides, addition of AAc has a positive, albeit small impact on nanoMIPs binding to all corresponding targets. This leads to a different conclusion as for peptides: that optimisation of the polymer composition is an unnecessary step for the synthesis of nanoparticles for imprinting of whole proteins. If the monomer composition is changed, then monomers will likely arrange themselves around different interaction sites on the protein's surface. Optimisation can however be useful if the task is to design MIPs that target different domains in a protein's structure, as an epitope imprinting strategy for generation of protein-selective MIPs will introduce a far narrower selectivity distribution, reducing the likelihood that a sample of MIPs will have affinity for a protein other than that containing the targeted epitope.

To verify whether MIPs produced here through solid-phase synthesis have appreciable affinity for corresponding templates, nanoMIPs were collected and tested using SPR (Table 2). All synthesised nanoMIPs demonstrated nanomolar affinities against the synthesised peptides, lower than those reported for nanoMIPs made in solution against similar-sized peptide sequences.^28^ It can therefore be concluded that for generation of MIPs for recognition of peptides, solid phase synthesis can afford excellent affinities with practically any ratio of the monomer composition tested here. Whilst all peptides tested demonstrated nanomolar affinities without optimisation, it should be noted that this is still too small a sample to say with confidence that a single polymer composition will work for all templates, and so there is still value in having this optimisation protocol should this standard composition fail for a more challenging analyte.

**Table 2:** Dissociation constants and sizes of MIPs synthesised using the solid-phase protocol with monomer composition 10

Analyte	*K*_D_ (nM)	Size (nm)
EPEGIYGVKKKC	2.40	192
EPEGIpYGVKKKC	1.90	201
SLNITSLGLRSLKEISDG	0.80	185
Amylase	0.34	285
Albumin	0.02	259
Trypsin	0.04	284

The results of this screening led to a conclusion that our aim – to develop a screening method for optimising nanoMIPs composition in a time-efficient manner – has been achieved.

## Conclusions

A screening procedure for optimisation of polymer compositions was designed utilising a 96-well filtration microplate, solid-phase synthesis combined with affinity separation, and a fluorescent reporter monomer. This allowed the simultaneous evaluation of 32 different monomer compositions in triplicate, generating information regarding affinity and selectivity introduced through a variety of monomers. Three peptides and three proteins were assessed using this method against a library of 10 functional monomer compositions. Modification of monomer ratios had an observable impact on the resulting MIPs affinity for peptide-immobilised solid phase, however very little influence was observed for protein-imprinted MIPs. The affinity of synthesised nanoMIPs confirmed using SPR was within the nanomolar range of dissociation constants, which is excellent for practical applications. The developed screening method will provide a useful tool for optimising compositions for enhanced affinity in the event that MIPs synthesised using the solid-phase protocol do not demonstrate appreciable binding.

## Conflicts of interest

There are no conflicts to declare.

## Supplementary Material
